# Analysis on the relationship between winter precipitation and the annual variation of horse stomach fly community in arid desert steppe, Northwest China (2007–2019)

**DOI:** 10.1111/1749-4877.12578

**Published:** 2021-08-10

**Authors:** Heqing HUANG, Ke ZHANG, Boru ZHANG, Shanhui LIU, Hongjun CHU, Yingjie QI, Dong ZHANG, Kai LI

**Affiliations:** ^1^ Key Laboratory of Non‐Invasive Research Technology for Endangered Species School of Ecology and Nature Conservation Beijing Forestry University Beijing China; ^2^ Chongqing Academy of Environmental Science Chongqing China; ^3^ Qinhuangdao Forestry Bureau Qinhuangdao China; ^4^ Office of Educational Administration Beijing Language and Culture University Beijing China; ^5^ Institute of Forest Ecology Xinjiang Academy of Forestry Urumqi China; ^6^ Mt. Kalamaili Ungulate Nature Reserve Changji Xinjiang China

**Keywords:** annual infection, arid desert steppe, *Gasterophilus*, Przewalski's horses, winter precipitation

## Abstract

*Gasterophilus* spp. have been found to be widespread in reintroduced Przewalski's horses in the Kalamaili Nature Reserve (Northwest China). However, data on the annual variation in *Gasterophilus* infections are lacking. To analyze the epidemiological features and determine the cause of the annual variation in *Gasterophilus* infections, we treated 110 Przewalski's horses with ivermectin and collected *Gasterophilus* larvae from fecal samples each winter from 2007 to 2019. All 110 Przewalski's horses studied were found to be infected by *Gasterophilus* spp., and a total of 141 379 larvae were collected. Six species of *Gasterophilus* were identified with the following prevalence: *G. pecorum* (100%), *G. nasalis* (96.36%), *G*. *nigricornis* (94.55%), *G. haemorrhoidalis* (56.36%), *G. intestinalis* (59.09%), and *G. inermis* (3.64%). The mean infection intensity of *Gasterophilus* spp. larvae in Przewalski's horses was 1285 ± 653. *G. pecorum* (92.96% ± 6.71%) was the most abundant species. The intensity of *Gasterophilus* spp. (*r* = –0.561, *P* < 0.046) was significantly correlated with winter precipitation. Our findings confirmed that, in the Kalamaili Nature Reserve, gasterophilosis is a severe parasitic disease in Przewalski's horses. Winter precipitation at the beginning of the year can indirectly affect the intensity and composition of *Gasterophilus* spp. in Przewalski's horses at the end of the year. Therefore, the water‐related ecological regulation should be carried out to help reduce the parasite infection of Przewalski's horses.

## Graphical abstract

The botfly infestation intensity of Przewalski’s horses were investigated for 13 years in Kalamaili Nature Reserve, China. The intensity and composition of botflies were affected by winter precipitation indirectly in Przewalski’s horses. The distribution of artificial water sources sites should be considered for decreasing the intensity of equines during the infection period of botflies.

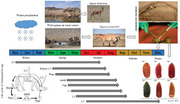

## INTRODUCTION

Przewalski's horses (*Equus przewalskii*) originate from the Hovd Basin of Western Mongolia and the eastern part of the Junggar Basin in China. The wild population became extinct in 1969, and the existing populations are the descendants of individuals captured 100 years ago (Mohr 1971). In order to restore wild populations of Przewalski's horses, The Foundation Reserves for the Przewalski's Horse (FRPH) was founded in 1980, and has sought to reintroduce Przewalski's horses in their native habitat (Wit *et al*. 2012).

In 1985, the Przewalski's horse was reintroduced in China. In 2001, 27 Przewalski's horses were released in the Kalamaili Nature Reserve (KNR), and the wild population has grown to around 230 in 2019. The KNR is also home to other ungulates, including the Mongolian wild ass (*Equus hemionus*), goitered gazelle (*Gazella subgutturosa*), and Argali sheep (*Ovis ammon*), and it provides winter pastures for populations of the nomadic Kazakhs (Wang *et al*. 2014). Currently, 3 equid species inhabit the reserve: the Przewalski's horse, the Mongolian wild ass, and overwintering domestic horses (*E. caballus*) (Huang *et al*. 2016).

*Gasterophilus* myiasis is a common disease of equine animals. The larvae of *Gasterophilus* species develop in the digestive tracts of equids, and the adults lay eggs on horses or grass. The generation time of these parasites is 1 year (Zumpt 1965). In China, 6 of the 9 known *Gasterophilus* species are present: *G. haemorrhoidalis, G. inermis, G. intestinalis, G. nasalis, G. nigricornis*, and *G. pecorum* (Xue & Zhao 1998). These species have all been reported to infect wild populations of Przewalski's horse in the KNR (Liu *et al*. 2016). Although the KNR reserve department drives all released Przewalski's horses back to fenced enclosures each winter, along with providing feed and administering ivermectin to ensure their health and reduce the impact of *Gasterophilus* spp. infection, the intensity of *Gasterophilus* spp. in the Przewalski's horse remains significantly higher than in the other 2 equids in the KNR (Huang *et al*. 2016). Previous studies have found that the source of *Gasterophilus* spp. in Przewalski's horses is the sympatric Mongolian wild asses and the domestic horses (Wang *et al*. 2014).

The KNR is located in arid desert steppe, with low precipitation, large evaporation, and no surface runoff, which made water source become an important factor restricting the range of wildlife activities in the reserve (Huang *et al*. 2016). Water is the source of life and has tremendous effects on animals. The shortage of water resources intensified the overlap of the activity space of Przewalski's horses and Mongolian wild asses around the water source (Huang *et al*. 2017). Frozen water sources and accumulated snow in winter will melt by the end of winter and the beginning of spring, which will affect the water volume and distribution of the water source, thus indirectly affect the activity range and overlap of equine animals in spring. Simultaneously, the annual spring migration of herders and their horses from the south (winter pasture) to the north (summer pasture) was also affected by water resources (Wang *et al*. 2014). In addition, there was a relatively fixed phenology and regularity in the occurrence of *Gasterophilus* spp. every year, and it mainly occurred in spring in the KNR (Wang *et al*. 2015). Therefore, we speculate that the winter precipitation will affect the parasite infection intensity of Przewalski's horses. The aim of the present study was to determine the factors that caused the variation in the epidemiological features of *Gasterophilus* spp. infection and to examine the effects of winter precipitation on the annual variation of infection in the released Przewalski's horses.

## MATERIALS AND METHODS

### Study area

The KNR (latitude: 44°36′–46°00′N, longitude: 88°30′–90°03′E, altitude: 600–1464 m) covers an area of approximately 17 000 km^2^. The vegetation in the KNR is sparse, mostly consisting of shrubs (*Anabasis brevifolia*, *Ceratoides laten*) and herbage (*Stipa caucaisica*). The area is characterized by a temperate continental arid climate, with a mean annual precipitation of 159 mm and a mean annual evaporation of 2090 mm and extremely little surface water (Zhang *et al*. 2015; Zang *et al*. 2017).

### Study samples

**Figure 1:**
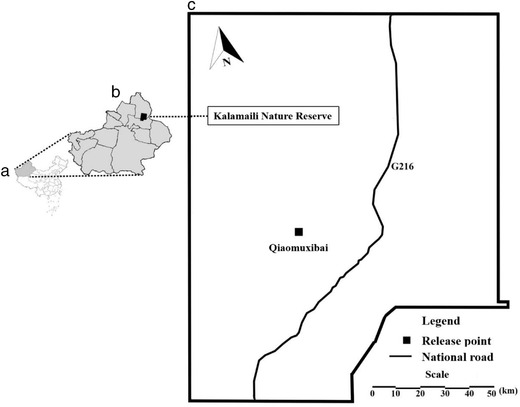
Location of the Kalamaili Nature Reserve, China.

Due to the annual freezing of water sources and shortage of plants in winter, the KNR reserve department drives all the released Przewalski's horses back to fenced enclosures of Qiaomuxibai (45°14.18′N, 89°02.75′E) (Fig. 1) in November and release them back into the wild in March of the following year, and our study samples were derived from the Przewalski's horses that were fenced each winter. However, some of the reintroduced populations have gradually adapted to the desert steppe and could follow the traces of the local indigenous species, the Mongolian wild ass, to explore a broader area to survive the harsh winter. Therefore, not all of the Przewalski's horses can be circled back to fenced enclosures every winter. In addition, although the released population was managed and cared for every winter, they were still wild animals in the process of rewilding, thus, kept high vigilance around humans, making it nearly impossible to study and had to be released. With all the reasons mentioned above, we carried out the experiment, whenever possible, with around 10 horses per year, and the horses selected varying in sex and age. According to the characteristics of wild horses, their ages were categorized into: <2 years (foal and yearling), 2–4 years (subadult), and >4 years old (adult).

### Collection of *Gasterophilus* spp. larvae

The high efficacy of ivermectin in controlling parasite infection has been established in horses (Bello 1996). In this study, Przewalski's horses were selected randomly during each winter (December to February) from 2007 to 2019. Individuals were kept in separate fenced enclosures and treated once with orally administered ivermectin (Beijing Wangfeng Farming Pharmaceutical Co., Ltd., Veterinary Drug) at the conventional dose of 0.2 mg/kg (Costa *et al*. 1998). The age and sex of each individual was recorded. After ivermectin treatment, all feces in each fenced area were collected separately at a frequency of 3 times daily (1000, 1400, 1800). The collection of feces continued to the fifth day (Dawson 2003) or until there were no larvae in feces for 3 consecutive days.

Tweezers were used to break up the horse feces and to separate the larvae from the feces. Larvae were stored in ethanol (75%), washed with phosphate‐buffered saline (PBS) or saline solution (0.9% NaCl), counted, and identified according to the identification method of the stomach bot fly larvae (Zumpt 1965; Li *et al*. 2018).

### Diversity

Shannon–Wiener diversity index (*H′*) and Pielou evenness index (*J*) were used to analyze the diversity of stomach bot flies in different years (Shannon 1948; Pielou 1966), and the relevant formulas are as follows:

(1) $$H^{\prime }=-\sum PilnPi\;\left(i=1,\;2,\;3,\;\ldots ,\;S\right)$$

(2) $$J=H^{\prime }/H_{\max}^{\prime }$$

In Equation (1), *Pi* is the ratio of the infection intensity of species *i* to the total intensity of *Gasterophilus* spp.; *S* is the number of species; $H_{\max}^{\prime }$ = ln*S*.

### Meteorological data collection

Winter precipitation data (December 1 to March 1 of the following year) for the relevant years were collected from the nearest observation point (45°22′N, 90°32′E) (CIMISS 2020). For example, the winter precipitation in 2007 is the accumulation of December 2006 plus January and February 2007, and 2019 is the accumulation of December 2018 and January plus February 2019.

### Statistical analysis

The prevalence, intensity, and abundance of infection were estimated according to the definitions provided by Margolis and Schad (1982). The relationships between the intensity of *Gasterophilus* spp. and winter precipitation were explored using Spearman's rank correlation. Tukey–Kramer tests were conducted to identify if the infection intensity of different ages and the proportion of *Gasterophilus* species significantly changed. The independent *t*‐test was used to examine the sex difference of infection intensity. The statistical analysis was performed using SPSS 20.0 and the figures were drawn by SigmaPlot 12.5. Significant differences were assumed when *P* ≤ 0.05.

## RESULTS

### Prevalence of *Gasterophilus* spp. larvae

Over the 13 years of this study, the overall prevalence of infection with *Gasterophilus* spp. larvae was 100% among the 110 examined Przewalski's horses. The collected *Gasterophilus* spp. larvae prevalences were as follows: *G. pecorum* was the most common species (found in 100% of horses) in the Przewalski's horse, followed by *G*. *nasalis* (96.36%), *G*. *nigricornis* (94.55%), *G. intestinalis* (59.09%), *G*. *haemorrhoidalis* (56.36%), and *G*. *inermis* (3.64%). The prevalence of *Gasterophilus* spp. larvae in Przewalski's horses with different sex and ages are also shown in Table 1.

**Table 1:** The prevalence of *Gasterophilus* spp. larvae in Przewalski's horses in the Kalamaili Nature Reserve

	Sex				Age							
	Male		Female		<2 year		2–4 year		>4 year		Total	
*Gasterophilus* spp.	No.	%	No.	%	No.	%	No.	%	No.	%	No.	%
*G. pecorum*	53	100	57	100	7	100	24	100	79	100	110	100
*G. nasalis*	50	94.34	55	96.49	4	57.14	24	100	77	97.47	106	96.36
*G. nigricornis*	51	96.23	53	92.98	4	57.14	24	100	76	96.20	104	94.55
*G. intestinalis*	28	52.83	37	64.91	5	71.43	16	66.67	44	55.70	65	59.09
*G. haemorrhoidalis*	26	49.06	36	63.16	4	57.14	18	75.00	40	50.63	62	56.36
*G. inermis*	2	3.77	2	3.51	1	14.29	0	0	3	3.80	4	3.64

The majority of the studied Przewalski's horses were infected by 4 (43.64%) or 5 (31.82%) species of *Gasterophilus*, whereas 19 (17.27%) Przewalski's horses were infected by 3 species, 4 (3.64%) Przewalski's horses were infected with 2 *Gasterophilus* species, and 3 (2.73%) Przewalski's horses were infected by all 6 species, only one (0.91%) was infected with 1 *Gasterophilus* species.

### Infection intensity of *Gasterophilus* spp. larvae

**Figure 2:**
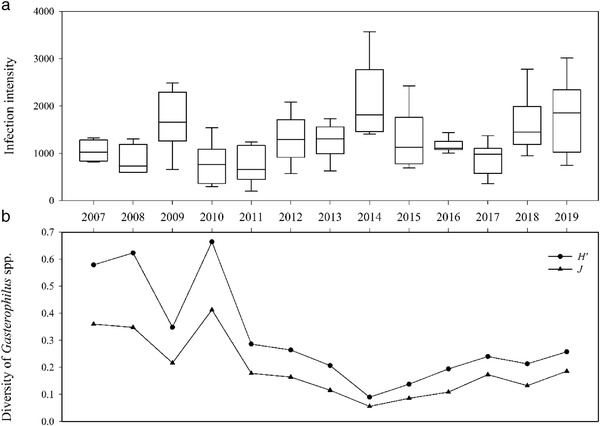
Infection intensity and diversity of *Gasterophilus* spp. larvae in Przewalski's horses from 2007 to 2019. (a) Infection intensity of *Gasterophilus* spp.; (b) diversity index.

A total of 141 379 larvae were collected from the 110 examined horses. The mean infection intensity of *Gasterophilus* spp. in the KNR was 1285.26 ± 652.94 (range: 173–3571) (Table 2). The years with the highest intensity of *Gasterophilus* spp. were 2009, 2014, and 2019 (Fig. 2a). *G*. *pecorum* was the most abundant species (92.96%, range: 61.30–100%), followed by *G*. *nigricornis* (3.46%, range: 0–29.09%), *G*. *nasalis* (2.68%, range: 0–13.77%), *G*. *haemorrhoidalis* (0.69%, range: 0–4.67%), *G. intestinalis* (0.21%, range: 0–2.47%), and *G*. *inermis* (range: 0–0.13%), which was only found in 2008, 2013, and 2016 (*F* = 11626.697, df = 5654, *P <* 0.001) (Table 2).

**Table 2:** The intensity and composition of *Gasterophilus* spp. larvae in Przewalski's horses in the Kalamaili Nature Reserve

		Intensity of *Gasterophilus* spp. (No. & %)								
Year	Przewalski's horses (No.)	*Gasterophilus* spp. (No.)			*G*. *pecorum*	*G*. *nigricornis*	*G. nasalis*	*G. haemorrhoidalis*	*G. intestinalis*	*G*. *inermis*
		Total	Range	Mean ± SD	*%* Mean ± SD					
2007	4	4184	817–1324	1046.00 ± 234.14	84.47 ± 3.38	6.99 ± 2.67	6.95 ± 2.32	1.42 ± 0.44	0.18 ± 0.13	0
2008	4	3369	592–1303	842.25 ± 329.34	82.57 ± 7.73	13.74 ± 7.21	1.31 ± 1.02	2.27 ± 0.80	0.09 ± 0.07	0.02 ± 0.04
2009	10	17108	633–2491	1710.80 ± 636.12	91.88 ± 3.97	2.82 ± 2.11	3.68 ± 1.83	1.54 ± 1.23	0.08 ± 0.15	0
2010	10	7674	300–1592	767.40 ± 433.27	79.45 ± 8.62	12.73 ± 8.42	7.33 ± 4.16	0.27 ± 0.23	0.22 ± 0.36	0
2011	10	7176	173–1247	717.60 ± 364.19	94.67 ± 2.99	0.98 ± 1.19	3.06 ± 2.00	1.18 ± 0.66	0.11 ± 0.12	0
2012	10	13291	558–2093	1329.10 ± 496.95	95.15 ± 2.81	1.27 ± 1.39	1.44 ± 0.80	1.75 ± 1.11	0.39 ± 0.36	0
2013	9	11514	630–1732	1279.33 ± 366.51	96.38 ± 1.61	0.48 ± 0.34	1.55 ± 1.10	1.41 ± 1.35	0.16 ± 0.18	0.02 ± 0.05
2014	5	10271	1404–3571	2054.20 ± 877.25	98.52 ± 1.46	0.59 ± 0.78	0.77 ± 0.79	0.04 ± 0.10	0.08 ± 0.11	0
2015	9	11506	689–2425	1278.44 ± 638.80	96.85 ± 3.09	1.56 ± 1.16	1.53 ± 2.03	0.04 ± 0.12	0.03 ± 0.08	0
2016	7	8186	1007–1441	1169.43 ± 145.25	96.35 ± 4.13	2.49 ± 3.97	0.83 ± 0.78	0.07 ± 0.10	0.25 ± 0.40	0.01 ± 0.03
2017	9	7846	357–1371	871.78 ± 327.05	94.88 ± 2.68	2.05 ± 1.84	2.37 ± 1.36	0	0.70 ± 0.84	0
2018	9	14354	947–2779	1594.89 ± 628.25	95.65 ± 1.80	2.33 ± 1.30	1.89 ± 1.44	0.05 ± 0.09	0.07 ± 0.12	0
2019	14	24900	724–3066	1778.57 ± 787.77	94.65 ± 3.90	2.95 ± 3.65	2.21 ± 2.00	0	0.20 ± 0.28	0
Total	110	141379	173–3571	1285.26 ± 652.94	92.96 ± 6.71	3.46 ± 5.11	2.68 ± 2.66	0.69 ± 1.00	0.21 ± 0.36	0 ± 0.02

The majority of the infected Przewalski's horses (78.18%) harbored between 500 and 2000 larvae, and 13.64% had more than 2000 larvae, only 8.18% had less than 500 larvae (Table 3). The average intensity of males (1310.34 ± 636.11) was similar to that of females (1261.95 ± 673.01) (*t* = 0.387, df = 108, *P* = 0.700). There were no significant differences in infection intensity among horses of different ages (*F* = 0.054, df = 2107, *P* = 0.948) (Table 3).

**Table 3:** Number and percentage of *Gasterophilus* spp. in Przewalski's horses grouped according to size of infection, sex, age

	Examined		Infection intensity
	No.	%	(Mean ± SD)
Size of infection			
0–500	9	8.18	365.56 ± 97.48
501–1000	29	26.36	760.41 ± 138.51
1001–1500	42	38.18	1241.21 ± 148.94
1501–2000	15	13.64	1720.40 ± 172.38
2001–2500	9	8.18	2276.22 ± 138.72
2501–3000	4	3.64	2744.25 ± 188.51
3001–3500	1	0.91	3066
3501–4000	1	0.91	3571
Sex			
Male	53	48.18	1310.34 ± 636.11
Female	57	51.82	1261.95 ± 673.01
Age			
<2 years	7	6.36	1313.29 ± 420.61
2–4 years	24	21.82	1247.25 ± 756.99
>4 years	79	71.82	1294.33 ± 642.34

### Diversity

With the exceptions of 2007, 2008, and 2010, the diversity of horse stomach bot flies in different years showed that the Shannon–Wiener diversity index (*H′*) was lower than 0.40, especially in 2014 (*H′* = 0.06) (Fig. 2b). Similarly, the Pielou evenness index (*J*) also showed similar characteristics.

### Correlation analysis between winter precipitation and *Gasterophilus* spp. intensities

**Figure 3:**
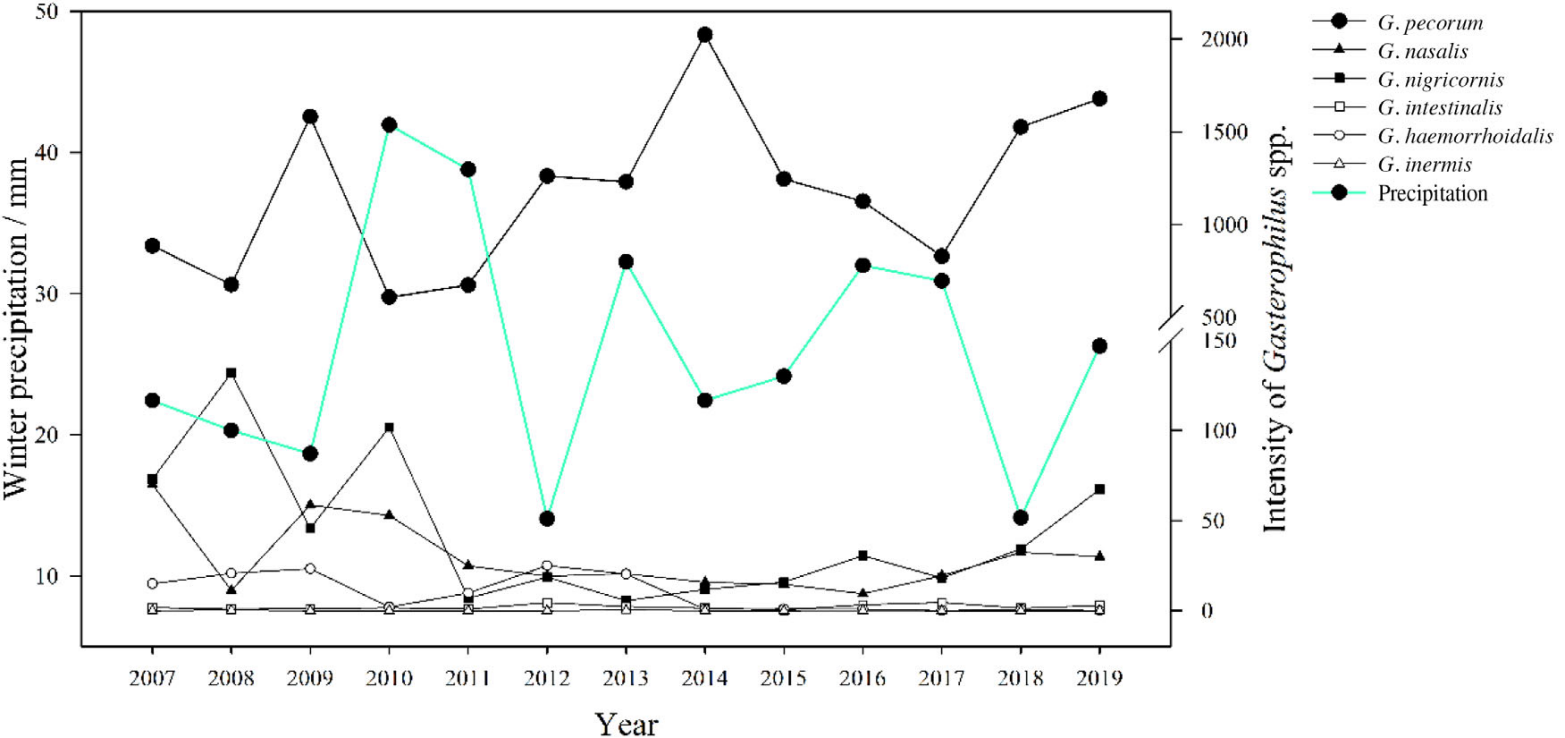
Winter precipitation and mean intensity of *Gasterophilus* species from 2007 to 2019.

**Figure 4:**
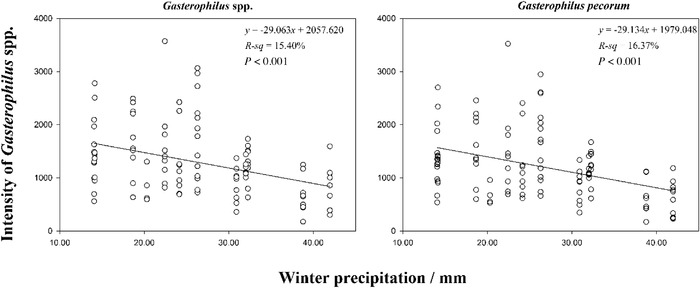
Relationship between winter precipitation and the intensity of *Gasterophilus* spp., *G. pecorum* in Przewalski's horses.

During the period of investigation, winter precipitation was highest in 2010 (Fig. 3). There were significant negative correlations between winter precipitation and the intensities of *Gasterophilus* spp. (*r* = –0.561, *n* = 13, *P* < 0.046), meaning that years with higher rainfall induced a lower *Gasterophilus* spp. population and vice‐versa. The increasing or decreasing tendencies of the intensities of *Gasterophilus* spp. and *G. pecorum* in relation to winter precipitation were demonstrated using a linear model (Fig. 4). *Gasterophilus* spp.: y=−29.063x+2057.620 (*R* ^2^ *=* 0.1540, *P* < 0.001); *G. pecorum*: y=−29.134x+1979.048 (*R* ^2^ *=* 0.1637, *P* < 0.001).

## DISCUSSION

The findings of the present survey revealed 100% prevalence and high intensity (1285) of *Gasterophilus* spp. infection in Przewalski's horses, which was considerably higher than that among equids in most parts of the world (Pandey *et al*. 1992; Agneessens *et al*. 1998; Studzińska & Wojcieszak 2009; Getachew *et al*. 2011; Ibrayev *et al*. 2015). We administered ivermectin to the Przewalski's horses almost every year, and these results indicated the importance of this parasite and that cross‐infection of *Gasterophilus* spp. was prevalent among the 3 types of equid inhabiting the KNR. In addition, there were no correlations between the infection intensity of *Gasterophilus* spp. larvae and gender and age of Przewalski's horses in this study.

In almost every year of the study, *G. pecorum* accounted for more than 90% of the total infection. This may be due to its unique behaviors. Although the adults of *Gasterophilus* spp. survive for less than 1 week, the eggs of *G. pecorum* can survive for several months under natural conditions (Zumpt 1965). *G. pecorum* is unique among *Gasterophilus* spp. in that it oviposits on grass (Chereshnev 1951). The number of eggs of *G. pecorum* (1300–2425) is higher than that of *G. intestinalis* (397–770), *G. nasalis* (489–518), *G. nigricornis* (330–350), and *G. haemorrhoidalis* (134–167) (Zumpt 1965). Chereshnev (1951) reported that the female *G. pecorum* oviposited 10–15 eggs on each plant, and one female can infect a large area of pasture. The high fecundity and oviposition on grass may thus increase infection probability and extend the time for infection. These features could make *G. pecorum* more efficient at infecting Przewalski's horses in sparsely vegetated areas.

The species composition of *Gasterophilus* may be affected by the host, the parasite, and/or environmental and management factors (Otranto *et al*. 2005). Furthermore, intraspecific effects may have a significant impact on parasite population regulation (Moller 2005). In general, the proportion of *G. pecorum* increased, whereas that of some other species decreased over time in the KNR. Annual ivermectin treatments and hostile interactions between *Gasterophilus* spp. due to interspecific competition may be factors that contributed to these trends.

The diversity of community reflects the characteristics of community structure (Watve & Sukumar 1995; Bechtel *et al*. 2015). Although there is an abundance of species of *Gasterophilus* spp. in the KNR, the dominant species, *G. pecorum*, accounts for a large proportion, resulting in a low diversity in this area. In recent years (2011–2019), the diversity indexes in the KNR have been in a low state (*H′* < 0.30, *J* < 0.20), which were lower than that of other regions in the world, such as South Africa (*H′* = 0.82, *J* = 0.75) (Krecek *et al*. 1989), Southern Italy (*H′* = 1.10, *J* = 0.68) (Otranto *et al*. 2005), and Eastern Turkey (*H′* = 0.72, *J* = 0.66) (Özdal *et al*. 2010). Each natural system is composed of many factors that can affect diversity (Frank 1993). The biological and environmental factors are conducive to the adaptation of *G. pecorum* to desert steppe, resulting in a large number of *G. pecorum* in the KNR. The stable selection stimulated the prevalence of a specific species and reduced community diversity.

In the KNR, the main source of *G. pecorum* in Przewalski's horses is the Mongolian wild ass (Wang *et al*. 2014). The KNR is located in a desert steppe, water resources are the most important factor limiting the range of wild equines. Therefore, Przewalski's horses and Mongolian wild asses had close contact in the KNR. Przewalski's horses appear to drink daily (Scheibe *et al*. 1998), and the oviposition sites of *G. pecorum* are often located near water sources (Liu *et al*. 2015). Lugauer found that established Przewalski's horses had longer daily travel distances than newly released individuals (Lugauer 2010). The released Przewalski's horses have not been completely restored to the wild; therefore, they are less sensitive to unnatural interference than Mongolian wild asses. Based on the fact that the cross‐spread of *G. pecorum* were between the 2 equine species, the interspecific adaptation difference in equines may explain the higher prevalence and density of *G. pecorum* population in this region, as well as the higher number in Przewalski's horses.

Meteorological factors may affect the population of *Gasterophilus* spp. (Sequeira *et al*. 2001), and in this regard, Pilo *et al*. (2015) found that the egg‐laying activity of *Gasterophilus* spp. was correlated with minimum air temperature and was reduced by rainfall in November and December. In this study, the infection intensity of *G. pecorum* was negatively related to winter precipitation. The availability of water is an important factor determining the distribution and habitat use of Przewalski's horses and Mongolian wild asses (Kaczensky *et al*. 2008). The variability in host distribution may alter parasite occurrence (Pickles *et al*. 2013). In the KNR, the shortage of water increases the interaction between Przewalski's horses and Mongolian wild asses (Huang *et al*. 2017), and the main infection period of *Gasterophilus* spp. is in spring (Wang *et al*. 2015). A higher winter precipitation may decrease the likelihood of sharing water sources and thereby reduce the niche overlap between Przewalski's horses and Mongolian wild asses, thus resulting in lower infection intensities of *G. pecorum* in Przewalski's horses.

Ecological regulation can be adopted to reduce the parasite infection of Przewalski's horses. Artificial water sources can be built in spring to reduce the aggregation of equines during the infection period of stomach bot flies, and help to reduce the cross‐spread of bot flies among equines. Considering the distribution of existing natural water sources in this region, new artificial water sources should be scattered, so as to avoid aggravating cross‐infection between animals. Especially in the years with less winter precipitation, control and management measures should be prepared in advance to reduce the cross‐infection of 2 wild equines during the transmission season of stomach bot flies and avoid acute occurrences of myiasis in equines.

## CONCLUSIONS

In this study, we confirmed that infection by *Gasterophilus* spp. in released Przewalski's horses is widespread in the KNR. We detected the occurrence of 6 species of *Gasterophilus* and a high intensity of infection in Przewalski's horses, and accordingly suggest that the KNR reserve department works to improve awareness of this major parasite. *G. pecorum*, the most abundant species, needs further investigation, allowing for better understanding of its infection cycle and to develop better control measures. In addition, winter precipitation can indirectly affect the intensity and composition of *Gasterophilus* spp. throughout the following year. Given that the local Mongolian wild asses and domestic horses appear to play important roles in transmitting *Gasterophilus* spp. infection to Przewalski's horses, it is necessary to take appropriate measures to reduce the cross‐infection between these equines.

## CONFLICT OF INTEREST

The authors declare no conflicts of interests.

## ETHICS STATEMENT

The study was performed in accordance with the relevant guidelines and regulations regarding animal welfare. All experimental protocols were approved by the Ethic and Animal Welfare Committee, Beijing Forestry University (Beijing, China).
